# The socioeconomic consequences of loneliness: Evidence from a nationally representative longitudinal study of young adults

**DOI:** 10.1016/j.socscimed.2024.116697

**Published:** 2024-03

**Authors:** Bridget T. Bryan, Katherine N. Thompson, Sidra Goldman-Mellor, Terrie E. Moffitt, Candice L. Odgers, Sincere Long Shin So, Momtahena Uddin Rahman, Jasmin Wertz, Timothy Matthews, Louise Arseneault

**Affiliations:** a Social, Genetic and Developmental Psychiatry Centre, Institute of Psychiatry, Psychology and Neuroscience, King's College London, 16 De Crespigny Park, London, SE5 8AF, UK; b Department of Sociology, College of Liberal Arts, Purdue University, 700 Mitch Daniels Blvd, West Lafayette, Indiana 47907-2059, USA; c Department of Public Health, School of Social Sciences, Humanities and Arts, University of California Merced, 5200 North Lake Road, Merced, 95343, USA; d Department of Psychology and Neuroscience, Duke University, 417 Chapel Drive, Durham, 27708, USA; e Department of Psychological Science, University of California Irvine, 4201 Social and Behavioral Sciences Gateway, Irvine, 92617, USA; f Chinese University of Hong Kong, Central Ave, Hong Kong, China; g Department of Psychology, University of Edinburgh, 7 George Square, Edinburgh, EH8 9JZ, UK; h School of Human Sciences, University of Greenwich, Park Row, London, SE10 9LS, UK

**Keywords:** Loneliness, Socioeconomic status, Employment, Employability, Social status, longitudinal

## Abstract

The negative health consequences of loneliness have led to increasing concern about the economic cost of loneliness in recent years. Loneliness may also incur an economic burden more directly, by impacting socioeconomic position. Much of the research to date has focused on employment status which may not fully capture socioeconomic position and has relied on cross-sectional data, leaving questions around the robustness of the association and reverse causation. The present study used longitudinal data to test prospective associations between loneliness and multiple indicators of social position in young adulthood, specifically, whether participants who were lonelier at age 12 were more likely to be out of employment, education and training (NEET) and lower on employability and subjective social status as young adults. The data were drawn from the Environmental Risk (E-Risk) Longitudinal Twin Study, a birth cohort of 2,232 individuals born in England and Wales during 1994–1995. Loneliness and subjective social status were measured at ages 12, 18 and 26. Employability and NEET status were assessed at age 18. Findings indicate that greater loneliness at age 12 was prospectively associated with reduced employability and lower social status in young adulthood. The association between loneliness and lower social status in young adulthood was robust when controlling for a range of confounders using a sibling-control design. Results also indicate that loneliness is unidirectionally associated with reduced subjective social status across adolescence and young adulthood. Overall, our findings suggest that loneliness may have direct costs to the economy resulting from reduced employability and social position, underlining the importance of addressing loneliness early in life.

## Highlights

• Loneliness is associated with multiple indicators of reduced socioeconomic position.
• Higher loneliness at age 12 precedes lower employability & social status at age 18.
• Association between loneliness & subjective social status in young adults is robust.
• Loneliness predicts later subjective status across adolescence and young adulthood.

## 1 Introduction

The health and economic consequences of loneliness have gained increasing attention from the public and policymakers in recent years with the US Surgeon General highlighting loneliness as a threat to economic prosperity in his recent advisory statement (US Department of Health and Human Services, 2023). As loneliness has been identified as a risk factor for mental health problems (Park et al., 2020), poor physical health (Petitte et al., 2015) and mortality (Holt-Lunstad et al., 2010), much of this concern has focused on the indirect economic costs arising from healthcare expenditure associated with loneliness (Mihalopoulos et al., 2020; Kung et al., 2021). However, loneliness may also exert an economic burden through a direct pathway from loneliness to socioeconomic outcomes. Loneliness has been linked to poor performance in education and employment (Lim et al., 2019) with lonely individuals more likely to be unemployed (Morrish and Medina-Lara, 2021) and less optimistic about their career prospects (Matthews et al., 2019). This suggests that experiencing loneliness may have consequences for individuals’ labour market participation and socioeconomic position. The association between loneliness and socioeconomic position is of particular concern in young adulthood, a period during which loneliness is prevalent (Victor and Yang, 2012; Qualter et al., 2015) and reduced social position has the potential to have long-lasting consequences for occupational functioning and health (Whitley et al., 2022).

Much of the research examining the link between loneliness and socioeconomic outcomes to date has focused on employment status (Morrish and Medina-Lara, 2021). While being employed is one key indicator of job market performance, relying solely on employment status to assess socioeconomic position risks over-simplifying experiences in the job market and overlooking the interlinked social and economic dimensions of social position (Savage et al., 2013). This is particularly true in young adulthood when individuals embark on their own occupational and educational pursuits and their social position becomes less bound to the position of their family. During this stage, educational and work careers often overlap and interact (Dougherty, 2022) as young people experience a range of combinations of post-secondary education, casual, part-time or full-time employment, and ‘time-out’, travel or ‘gap years’ (Vogt, 2018). As such, being out of employment or in a low-status occupation in young adulthood may not fully reflect an individual's job market prospects across their working life.

Young adults' potential to perform well in the job market, or their employability, may be a more reliable indicator of their socioeconomic position than employment status. Employability is the capacity of an individual to gain meaningful and sustainable employment and depends on the knowledge, skills and attitudes a person brings to the job market, as well as the conditions of the labour market in which they pursue employment (Hillage and Pollard, 1998). An individual's employability is shaped by personal assets including technical skills, ‘soft’ skills, qualifications, work experience and job seeking behaviour (McQuaid and Lindsey, 2005). Subjective social status scales, in which individuals assess their position on the socioeconomic ‘ladder’, offer another alternative for assessing social position in ways that may matter for a successful transition to young adulthood. Evidence suggests that subjective ratings of social status capture more subtle, proximal aspects of social hierarchies (Demakakos et al., 2008; Grace, 2017) with young people's ratings influenced by a range of dimensions of their social position including educational achievements, positive elements of their job (Nielsen et al., 2015), their mental health, employment status and contact with the criminal justice system (Rivenbark et al., 2020). Research using age-appropriate indicators of young people's social position is needed to better understand the link between loneliness and social position and assess the direct economic costs of loneliness.

Further, the mechanisms underlying the association between feeling lonely and reduced socioeconomic position are unclear with a range of individual and situational factors potentially explaining this association. Children who are lonely during their school years may experience social and academic difficulties, such as bullying and school refusal, that can result in them leaving school with lower qualifications (Matthews et al., 2023) and disadvantaged in the job market. Mental health problems linked with loneliness may also drive this association. Tests of the association between loneliness and social status while accounting for poor mental health, functioning and unmeasured aspects of social position shared within families will provide new insights into the robustness of the association. Sibling designs offer a unique opportunity to test the association while adjusting for a range of unobservable aspects of family and household social position that are shared by individuals from the same family. Further control for objective indicators of social position such as educational attainment and employment status will also help test the robustness of the association.

Additionally, as most existing research on the topic has utilised cross-sectional data (Morrish and Medina-Lara, 2021), the temporality of the association is not well understood. While academic and mental health difficulties associated with loneliness may impede lonely individuals’ employment prospects, in light of the importance of employment for identity and social inclusion (Correa-Velez et al., 2013), being unemployed may also lead to feelings of isolation and rejection. A small number of longitudinal studies point to prospective associations both from loneliness to unemployment (Morris, 2020, von Soest et al., 2020) and from unemployment to loneliness (Buecker et al., 2021). However, they do not adjust for prior history of job market performance or loneliness, raising the issue of reverse causation. Prospective longitudinal research focused on young adults as they enter the job market will provide insight into this association and facilitate the identification of young people at risk of poor work outcomes.

In this study, we investigated the associations between loneliness and multiple indicators of social position in young adulthood in a nationally representative British longitudinal cohort. We aimed to (1) test whether loneliness is associated with social position using measures of NEET status, employability and subjective social status in young adulthood adjusting for correlates of loneliness, (2) test the robustness of the association between loneliness and social status using a sibling-control design, and (3) estimate the direction of the associations between loneliness and subjective social position using longitudinal analyses.

## 2 Method

### 2.1 Participants

Participants were members of the Environmental Risk (E-Risk) Longitudinal Twin Study, which tracks the development of a cohort of 2,232 British twins. The sample was drawn from a larger birth register of twins born in England and Wales in 1994–1995 (Trouton et al., 2002). Full details of the sample are reported elsewhere (Moffitt and E-Risk Study Team, 2002). Briefly, the E-Risk sample was constructed in 1999–2000, when 1,116 families (93% of those eligible) with same-sex 5-year-old twin pairs participated in home-visit assessments. This sample comprised 56% monozygotic (MZ) and 44% dizygotic (DZ) twin pairs. Sex was evenly distributed within zygosity (49% male). 90% of participants were of White ethnicities.

Follow-up home visits were conducted when the children were aged 7 (98% participation), 10 (96%), 12 (96%), and at 18 years (93%). 2,066 individuals participated in the E-Risk assessments at age 18. The proportions of MZ twin pairs (56%) and male (47%) twins who participated at age 18 were almost identical to those in the original sample at age 5. All interviews were conducted after participants’ 18th birthday; the average age of the twins at the time of the assessment was 18.4 years (SD = 0.36). There were no differences between those who did and did not take part at age 18 in terms of parental socioeconomic status (SES) assessed when the cohort was initially defined (χ^2^(2, N = 2,232) = 0.86, *p* = 0.65), age-5 intelligence quotient (IQ) scores (t(2,208) = 0.98, *p* = 0.33), or age-5 emotional or behavioural problems (t(2,230) = 0.40, *p* = 0.69 and t(2,230) = 0.41, *p* = 0.68, respectively). Home visits at ages 5, 7, 10, and 12 years included assessments with participants as well as their mother or primary caretaker. The home visit at age 18 included interviews only with the participants.

An online follow-up was conducted when participants were aged 26. All E-Risk participants were invited to complete the web-based Social Media and Social Mobility (SM2) survey taking approximately 15–20 minutes. Questions covered usage of social media and digital technology, interpersonal trust, political engagement, mental health, employment, and beliefs about social mobility. A total of 1,632 participants completed the survey, representing 73.1% of the original cohort and 76.6% of those who took part in the age-18 home visits. The proportion of male (42.3%) and low-parental SES study members (31.4%) who completed the survey was similar to that at the age 18 visits (47% male, 33% low SES). Study members who participated in the age 26 survey reported lower loneliness at age 12 (mean = 0.46, SD = 0.85), than those who did not participate (mean = 0.54, SD = 0.91), but the difference was not significant (p > 0.05).

The study sample's neighbourhoods represent the full range of socioeconomic conditions in Great Britain. Fig. S1 illustrates that E-Risk study families' addresses mirror the deciles of the UK government's 2015 Index of Multiple Deprivation, which ranks British neighbourhoods in terms of relative deprivation at an area level of approximately 1,500 residents. Approximately 10% of the E-Risk study cohort fills each of the index's 10% bands, indicating that the cohort accurately represents the distribution of deprivation in the United Kingdom.

The Joint South London and Maudsley and the Institute of Psychiatry Research Ethics Committee approved each phase of the study. Parents gave informed consent and twins gave assent between 5 and 12 years. Twins gave informed consent at ages 18 and 26.

### 2.2 Measures

#### 2.2.1 Loneliness

A measure of loneliness at age 12 was derived using three items from the Children's Depression Inventory (Kovacs, 1992). Each item was presented as a set of three statements, and participants were instructed to select the statement that described them best: (1) “I do not feel alone,” “I feel alone many times” or “I feel alone all the time”; (2) “I have plenty of friends,” “I have some friends but I wish I had more” or “I do not have any friends”; and (3) “Nobody really loves me,” “I am not sure if anybody loves me,” “I am sure that somebody loves me.” Items were coded 0–2 and item 3 was reverse coded. Items were summed to produce a scale from 0 to 6 (mean = 0.48, SD = 0.86, ω = 0.49) where higher scores indicate higher feelings of loneliness. This scale was used for all analyses, except when calculating mean loneliness scores, when age 12 scores were rescaled to produce a 0 to 8 scale to facilitate comparison with the scale used to measure loneliness at ages 18 and 26 (mean = 0.64). While internal consistency was low, this measure has been shown to perform similarly to more well-validated loneliness measures, in its pattern of associations with known correlates of loneliness such as victimisation (Matthews et al., 2022a). Further, while extracted from an instrument designed to assess depression, the constituent particular items are very similar in content to items used in the Children's Loneliness Scale, which is considered the gold standard for assessing loneliness in children and young adolescents (Maes et al., 2017).

Loneliness was measured at age 18 and 26 using four items from the UCLA Loneliness Scale, Version 3 (Russell, 1996): “How often do you feel that you lack companionship?”, “How often do you feel left out?”, “How often do you feel isolated from others?” and “How often do you feel alone?” A very similar short form of the UCLA scale has previously been developed for use in large-scale surveys, and correlates strongly with the full 20-item version (Hughes et al., 2004). At ages 18 and 26, the scale was administered as part of a self-complete questionnaire. The items were rated “hardly ever” (0), “some of the time” (1), or “often” (2). Items were summed to produce a total loneliness score from 0 to 8 (at age 18: mean = 1.57, SD = 1.94, ω = 00.84; at age 26: mean = 2.43, SD = 2.27, ω = 00.85).

The correlation between loneliness measured at age 12 and at age 18 was *r* = 0.25, and between age 18 and age 26 was *r* = 0.40. The pattern of associations between the loneliness and mental health conditions and social isolation were similar for both the age 12 loneliness measure and the UCLA scale used at age 18 (Table S1). Loneliness scores at ages 18 and 26 did not differ by parental SES; at age 12, participants from lower SES parents reported higher loneliness than those from higher SES backgrounds (low mean = 0.74, middle mean = 0.65, high mean = 0.51; *p* = 0.001; full detail in Table S2). Age-12 and -18 loneliness scores did not vary by sex; at age 26, female participants reported higher loneliness than male participants (females mean = 2.55, males mean = 2.28; *p* = 0.03; full detail in Table S2).

#### 2.2.2 Socio-economic indicators

*NEET status.* At age 18, participants were classified as NEET if they reported that they were not studying, working in paid employment, or pursuing a vocational qualification or apprenticeship training. At age 18, participants were queried to ensure that NEET status was not simply a function of being on summer holiday or being a parent. This operationalisation of NEET status aligns with classifications used by the UK Office for National Statistics and the International Labor Organization (Office for National Statistics, 2013). At age 18, approximately one in nine E-Risk study participants (11.6%, n = 239) were NEET (Goldman-Mellor et al., 2016).

*Employability.* We collected information on 7 indicators of young adults' education, employment history and work-related self-perceptions which we used to construct an index of employability at age 18. Participants' highest educational attainment was rated on a four-point scale: no qualifications (0), GCSE at grades D-G (1), GCSE at grades A*-C (2), and A Levels (3). Participants' employment history was rated on a three-point scale: never employed (0), previously employed (1), and currently employed (2). Participants were also interviewed about their perceived job preparedness, specifically, whether they possessed professional/technical skills (e.g., computer programming, sales skills) and ‘soft’ skills (behavioural competencies such as teamwork and decision-making). We also interviewed participants about their optimism about their career prospects (e.g., “the job market is usually good to people like you”), their attitudes towards work (e.g., “Having a job means more to me than just the money”) and perceptions of barriers to gaining employment. Each work-related self-perception measure was a score derived from summing the participant's responses to individual items. Measures are described in more detail in Table S3.

We performed an unrotated exploratory factor analysis of the above measures to identify the underlying construct of employability. Bartlett's Test of Sphericity (χ^2^(21) = 1457.45, p < 0.001) and the Kaiser-Meyer-Olkin statistic (KMO = 0.63) indicated that the data were suitable for factor analysis (Bartlett, 1950; Kaiser, 1974). The results of the factor analysis suggested a one-factor solution was suitable, with only one factor producing an eigenvalue greater than one (Table 1), and the scree plot showing a clear inflection point at the second factor (Fig. S2). As such, we retained one factor labelled ‘employability’. We estimated factor scores using the regression method to create a continuous measure of employability.

**Table 1:** Factor loadings, uniqueness and eigenvalues for exploratory factor analysis.

Variable	Factor loading			
	1	2	3	Uniqueness
Educational achievement	**0.41**	−0.26	0.17	0.74
Work history	**0.30**	0.15	−0.21	0.85
Job preparedness – professional skills	**0.36**	0.25	0.19	0.77
Job preparedness – soft skills	**0.56**	0.20	0.04	0.65
Career optimism	**0.62**	−0.22	0.02	0.57
Factors hurting job chances	**−0.35**	0.18	0.28	0.77
Attitudes to work	**0.20**	0.33	−0.08	0.85
Eigenvalue	**1.24**	0.38	0.20	

*Subjective social status* was measured at ages 12, 18 and 26 using an adapted version of the MacArthur Scale of Subjective Social Status (Goodman et al., 2001). Participants were shown an image of a ladder with 5 rungs and told the following: “this ladder represents how things are in the United Kingdom. At the top of the ladder are all the people who have the best jobs, lots of money, live in nice places, and go to the best schools. At the bottom of the ladder are those people who don't have enough money, don't live in a nice place, and might not have a job.” At age 12, participants were then asked, “Now think about your family—where would they be on the ladder?” At ages 18 and 26, the wording was adapted to be more age-appropriate: “Now think about *you*—where would *you* be on the ladder?” Participants were instructed to select which rung best represented their position, with the lowest rung (coded 1) representing “poor” and the highest rung (5) representing “rich.” Participants, on average, rated their social status scores as 3.51, 3.12, and 3.06 at ages 12, 18 and 26, respectively. The correlations between social status ratings between sweeps were small to moderate (Table 2).

**Table 2:** Associations among indicators of social position as indicated by Pearson's correlation coefficients.

	Parental SES	NEET status (age 18)	Employability (age 18)	Subjective social status (age 12)	Subjective social status (age 18)	Subjective social status (age 26)
**Parental SES**	1					
**NEET (age 18)**	−0.22	1				
**Employability (age 12)**	0.28	−0.38	1			
**Subjective social status (age 12)**	0.15	−0.03	0.08	1		
**Subjective social status (age 18)**	0.33	−0.23	0.33	0.23	1	
**Subjective social status (age 26)**	0.22	−0.15	0.24	0.11	0.32	1

All p < 0.01.

#### 2.2.3 Covariates

*Age-5 covariate.* Parental SES was measured using a standardised composite of household income, parents' education, and parents’ occupation when participants were aged 5. Parental SES was split into tertiles that grouped the sample into low, medium and high parental SES.

*Age-12 covariates.* Childhood covariates were grouped into two domains: mental health and risky behaviours. Mental health was indicated by depression symptoms, anxiety symptoms and neuroticism. Depression symptoms were measured using the depression subscale of the Child Behaviour Checklist for mothers (Achenbach, 1991). Anxiety was measured using child self-report using the 10-item Multidimensional Anxiety Scale for Children (MASC; March et al., 1997). Neuroticism was assessed using an adapted form of the Big Five Inventory completed by interviewers after the home visit (John and Strivastava, 1999). Risky behaviour was indicated by mothers' reports of whether the participants smoked tobacco or drank alcohol without their parents’ permission.

*Age-18 covariates.* Young adulthood covariates were similarly grouped into mental health and functioning domains. Participants' symptoms of major depressive disorder and generalised anxiety disorder were assessed using a structured interview according to DSM-IV criteria using the Diagnostic Interview Schedule (DIS; Robins et al., 1995). Neuroticism was measured using an adapted form of the Big Five Inventory completed by interviewers after the home visit (John and Strivastava, 1999). Poor functioning at age 18 was indicated by early parenthood, criminal offending and alcohol use. Early parenthood was assessed by self-reports of any sexual contact that had resulted in childbirth prior to the age-18 interview. Participants' criminal offending history was obtained via linkage to the UK Ministry of Justice's Police National Computer (see Motz et al., 2020 for more detail). Alcohol use disorder symptoms were assessed using the Diagnostic Interview Schedule for alcohol use disorder (DIS; Robins et al., 1995).

### 3.3 Statistical analyses

#### 3.3.1 Is loneliness associated with lower social position?

We tested whether age-18 loneliness was associated with concurrent NEET status, employability and social status separately using a series of logistic and linear regression models planned a priori. As a first step, we tested whether age-18 loneliness was associated with each outcome, adjusting only for parental SES and sex. To test whether the effect of loneliness on each outcome was accounted for by concurrent mental health problems and poor functioning, we added measures of depression, anxiety and neuroticism to the model in step 2, and then early pregnancy, alcohol use disorder and criminal offending in step 3. In step 4, we assessed the robustness of the relationship between age-18 loneliness and concurrent indicators of social position by adjusting for age-12 loneliness.

We then tested whether loneliness preceded age-18 indicators of socioeconomic position in a univariate model using age-12 loneliness as the independent variable. In step 6, we added age-12 depression, anxiety and neuroticism into the model, and added age-12 tobacco smoking and alcohol use to the model in step 7. Standard errors in all models were adjusted for the clustering of twin observations within families.

#### 3.3.2 Do lonelier individuals have lower subjective social status when controlling for objective indicators of social status?

To test the robustness of the association between loneliness and subjective social status, we used a sibling-control method to compare the reports of twin pairs living in the same household. We did this by computing within-twin pair difference scores for loneliness and for social status and testing the association between these difference scores in a regression framework. This approach holds household and family socioeconomic status constant by design. As such, differences within twin pairs cannot be explained by features of the household or family-wide social status, but instead are accounted for by genetic and environmental factors unique to individual twins. Thus, correlation between within-pair differences in loneliness and within-pair differences in their ratings of their social status indicates an effect of loneliness that is independent of individuals’ actual household or childhood family social status. To further test whether these differences were explained by differences in educational achievement or employment status, we controlled for within-twin pair difference scores in education and NEET status as a second step. As a third step, we added twin differences in employability scores to the model to test whether the association was explained by differences in employability.

#### 3.3.3 Bidirectional associations of loneliness and subjective social status across early adolescence and young adulthood

To describe changes in loneliness and subjective social status across time, we computed cross-wave difference scores for both loneliness and social status for the periods from age 12 to age 18, from age 18 to 26 and from age 12 to age 26. We calculated correlation coefficients to examine whether changes in loneliness and social status were associated across time.

To assess the direction of the association between loneliness and social status at age 12, 18 and 26, we used the random-intercept cross-lagged panel model (RI-CLPM; Hamaker et al., 2015). This enables us to assess change that occurs for each individual from one time point to the next (within-person effects) while accounting for stable trait-like differences between individuals (between-person effects). The RI-CLPM simultaneously estimates cross-sectional correlations between loneliness and social status at each time point, how loneliness and social status fluctuate from one time point to the next (autoregressive paths), and the within-person bidirectional associations between loneliness and social status across time (cross-lag paths)*.* We imposed equality constraints on the autoregressive and cross-lag paths to determine whether a more parsimonious model representing consistent change over time would adequately fit the data. We handled missing values using Full Information Maximum Likelihood (FIML) and accounted for the non-independence of twin observations by calculating robust standard errors.

Correlation, regression, and twin analyses were conducted in Stata 17 (StataCorp, 2019). The RI-CLPM was estimated in R v4.0.3 (R Core Team, 2022) using lavaan v0.6-10 (Rosseel, 2012). The analysis plan was pre-registered at sites.duke.edu/moffittcaspiprojects/projects_2022/sites.duke.edu/moffittcaspiprojects/projects_2022/ and analysis code is available at github.com/bridgetbryan/loneliness-socioeconomic-consequences.

## 4 Results

### 4.1 Is loneliness associated with lower social position?

Lonelier 18-year-olds were more likely to be NEET, less employable and rate themselves as having lower social status in univariate analyses (Table 3, model 1), and when accounting for concurrent mental health problems and poor functioning (models 2 and 3). When adjusting for loneliness at age 12 (model 4), young adults’ loneliness remained associated with lower employability and lower subjective social status; standardised regression coefficients indicated modest associations. The association with NEET status fell marginally below the significance threshold in the final model.

**Table 3:** Hierarchical regression analyses modelling the association between loneliness and age 18 occupational functioning.

	NEET status					Employability					Subjective social status				
	OR	(SE)	95% CI		*p*	b	(SE)	95% CI		*p*	b	(SE)	95% CI		*p*
**Loneliness (age 18)**															
Model 1: Adjusted for parental SES and sex	1.18	(0.04)	1.10	1.26	*<0.001*	−0.12	(0.01)	−0.13	−0.10	*<0.001*	−0.08	(0.01)	−0.10	−0.06	*<0.001*
Model 2: Adjusted further for age-18 mental health problems	1.09	(0.04)	1.01	1.18	*0.02*	−0.09	(0.01)	−0.11	−0.07	*<0.001*	−0.06	(0.01)	−0.08	−0.04	*<0.001*
Model 3: Adjusted further for age-18 functioning	1.10	(0.05)	1.02	1.19	*0.02*	−0.09	(0.01)	−0.11	−0.07	*<0.001*	−0.06	(0.01)	−0.08	−0.04	*<0.001*
Model 4: Adjusted further for age-12 loneliness	1.08	(0.05)	1.00	1.18	*0.06*	−0.08	(0.01)	−0.10	−0.05	*<0.001*	−0.05	(0.01)	−0.08	−0.03	*<0.001*
**Loneliness (age 12)**															
Model 5: Adjusted for parental SES and sex	1.28	(0.09)	1.11	1.48	*<0.001*	−0.18	(0.02)	−0.23	−0.14	*<0.001*	−0.10	(0.02)	−0.14	−0.05	*<0.001*
Model 6: Adjusted further for age-12 mental health problems	1.16	(0.10)	0.99	1.36	*0.07*	−0.13	(0.02)	−0.18	−0.09	*<0.001*	−0.07	(0.02)	−0.12	−0.03	*<0.01*
Model 7: Adjusted further for age-12 risky behaviour	1.16	(0.10)	0.99	1.36	*0.07*	−0.13	(0.02)	−0.18	−0.09	*<0.001*	−0.07	(0.02)	−0.12	−0.03	*<0.01*

*Note:* Mental health problems at age 12 and age 18 indicated by symptoms of major depressive disorder, symptoms of generalised anxiety disorder and neuroticism. Age-18 functioning indicated by early parenthood, criminal offending and alcohol use. Age 12 risky behaviour indicated by tobacco smoking and drinking alcohol without parents' permission.

When examining whether loneliness predicted later social position, we observed that participants who felt lonelier at age 12 were more likely to be NEET, have lower employability scores and rate themselves as having lower social status six years later as they entered adulthood (Table 3, model 5). When accounting for mental health symptoms and risky behaviour at age 12 (model 7), the associations between early adolescent loneliness remained significantly associated with reduced employability and social status in young adulthood. As in the cross-sectional analyses, these associations were modest.

Results did not significantly differ between participants with low, medium or high parental SES or those with high or low loneliness at ages 12 or 18. Similarly, results did not vary between male and female participants, except for the association between age-12 loneliness and employability in young adulthood where girls who felt lonelier at age 12 had lower employability at age 18 (b = −0.23, p < 0.001), than their similarly lonely male peers (b = −0.14, p < 0.001).

#### 4.1.1 Do lonelier 18-year-olds have lower subjective social status when controlling for objective indicators of social status?

**Fig. 1:**
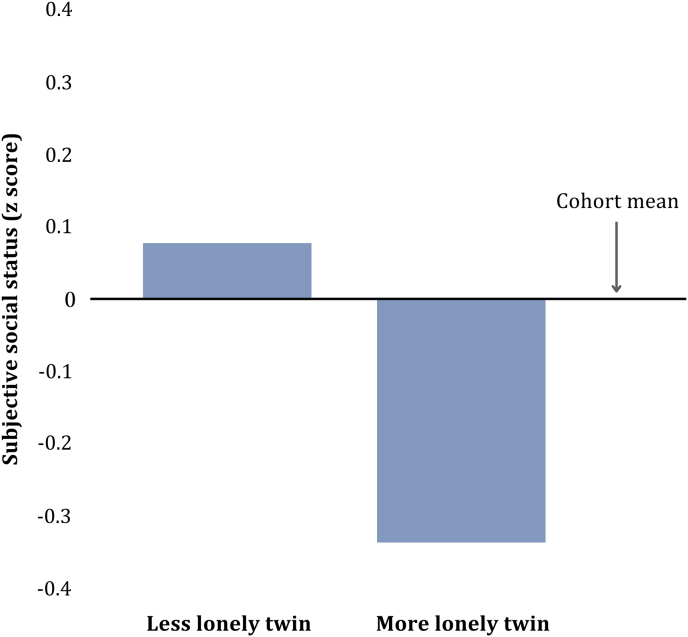
**Standardised mean subjective social status scores comparing less lonely vs. more lonely twins among 132 twin pairs discordant for loneliness.** Restricted to twin pairs discordant for loneliness by four or more points at age 18. (N = 132 twin pairs).

Among cohabiting twin pairs, within-pair differences in loneliness were significantly associated with differences in subjective social status (Table 4, model 1) indicating that lonelier individuals perceived themselves to have lower social status than their less lonely co-twin living in the same household. That is, loneliness was associated with lower social status ratings after actual childhood socioeconomic status and current household social status were held constant by design. This association remained significant when analyses controlled further for twin differences in educational achievement, NEET status and employability scores (Table 4, model 3). As such, even among pairs of twins matched on childhood family and current household social status, educational achievement, employment and employability, lonelier twins rated their social status as lower than their less lonely co-twin. This is illustrated in Fig. 1 which shows that in a subgroup of twin pairs discordant on loneliness, lonelier twins rated their social status significantly lower (mean = −0.33, SD = 1.24) than their less-lonely co-twin (mean = 0.07, SD = 1.06; t(261) = 2.88).

**Table 4:** Regression analyses modelling the association between within-pair differences in loneliness and subjective social status at age 18.

Difference scores	Model 1			Model 2			Model 3		
	b	SE	Sig	b	SE	Sig	b	SE	Sig
Loneliness	−0.04	0.01	*<0.001*	−0.04	0.01	*<0.001*	−0.04	0.01	*<0.01*
Education achievement				−0.08	0.04	*0.03*	−0.13	0.04	*0.001*
NEET status				−0.41	0.08	*<0.001*	−0.30	0.08	*<0.001*
Employability							0.16	0.04	*<0.001*
N (twin pairs)	808			804			801		

Restricted to twins living in the same household at age 18.

#### 4.1.2 Do loneliness and social status predict each other across early adolescence and young adulthood?

Participants' ratings of their position in the social hierarchy declined at each age, although their scores remained close to the middle of the social hierarchy (Table 5). Conversely, participants’ loneliness scores increased at each time point. Changes in loneliness scores between each wave were negatively associated with changes in social status across the same time period such that increasing levels of loneliness were associated with decreasing social status ratings (*r* = −0.16 to −0.12, all *p* < 0.001).

**Table 5:** Mean loneliness and subjective social status at each time point.

Variable			F		
	Mean	(SD)	Age 12-18	Age 18-26	Age 12-26
**Loneliness (0–8 scale)**			**F**_**1,1013**_	**F**_**1,892**_	**F**_**1,890**_
Age 12	0.64	1.15	82.74***		
Age 18	1.57	1.94		247.43***	
Age 26	2.43	2.27			26.86***
**Subjective social status (1–5 scale)**			**F**_**1,1008**_	**F**_**1,892**_	**F**_**1,881**_
Age 12	3.51	0.65	83.38***		
Age 18	3.12	0.74		138.84***	
Age 26	3.06	0.76			13.43***

***p < 0.001.

**Fig. 2:**
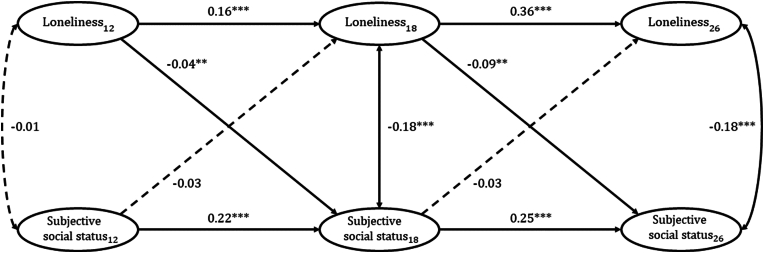
**Within-person longitudinal associations between loneliness and subjective social status across ages 12, 18 and 26 using the random-intercept cross-lagged panel model.** Values on single-headed arrows are standardised partial regression coefficients. Values on double-headed arrows between variables at the same timepoint are correlation coefficients. Nonsignificant paths are indicated by dashed arrows. Significant paths are indicated by solid arrows. Subscript numbers indicate timepoint of assessment. Autoregressive and cross-lag paths were constrained to be equal across time. Full constrained and unconstrained models including between-person associations are shown in Fig. S2. (N = 2,195) **p < 0.01, ***p < 0.001.

Constraining the autoregressive and cross-lagged paths did not reduce the model fit of the RI-CLPM and the constrained model was retained. The stable group-level between-person association showed that, on average, lonelier individuals rated themselves as having lower social status (*r* = −0.80, p < 0.01, Fig. S3). When examining the individual-level associations between loneliness and social status across ages 12, 18 and 26, we found that the autoregressive effects of loneliness and social status were significant across the three waves, indicating their stability across the three time points (Fig. 2, full detail in Supplement D). Loneliness and social status were cross-sectionally associated at age 18 and 26, but not at age 12. Results for the lagged effects from age 12 to 18 indicated that while participants' ratings of their social status at age 12 did not influence how lonely they felt at age 18, an increase in loneliness (relative to each individual's average level of loneliness) was associated with reduced social status at age 18 with a small effect. When examining the associations between social status and loneliness at ages 18 and 26, results similarly show that loneliness at 18 years old had a moderate negative effect on social status ratings when participants were in their mid-twenties. Participants' social status ratings at age 18 did not influence loneliness in their mid-twenties.

## 5 Discussion

While there are growing concerns around the economic impact of loneliness, much of the research on the topic has focused on healthcare costs indirectly associated with loneliness (Leigh-Hunt et al., 2017). Our findings indicate that loneliness may have enduring consequences for socioeconomic position, pointing to an additional direct pathway through which loneliness exerts an economic burden on both individuals and society more broadly. Our results show that loneliness in early adolescence is prospectively associated with multiple indicators of reduced socioeconomic position in young adulthood and that the association between loneliness and subjective social status in young adulthood is robust and unidirectional. Altogether, our findings suggest that addressing loneliness early may have the potential to have long term benefits for individuals’ socioeconomic outcomes, and in turn, broader economic benefits associated with greater productivity across the working-age population.

Our findings build on previous research that has identified cross-sectional associations between loneliness and poor employment outcomes (Matthews et al., 2019; Morrish and Medina-Lara, 2021) by showing a prospective association between loneliness and multiple indicators of social position. Reduced social position and labour market preparedness associated with loneliness in young adulthood may have enduring consequences for lonely individuals throughout their lives and lead to sustained downward social mobility and productivity costs for the economy more broadly (Mihalopoulos et al., 2020). As such, addressing loneliness may improve the socioeconomic prospects of young people, in addition to benefitting health and wellbeing. Improving young people's socioeconomic outcomes may also have broader economic benefits resulting from improved work engagement and productivity.

Our results also show that the association between loneliness and lower subjective social status is robust when controlling for a range of objective and subjective indicators of social status, indicating that the association is driven by factors specific to lonely individuals. The sibling control analyses show that even when twin pairs were matched on childhood family status and household status by design, and when adjusting for education, employment status and employability, the association between loneliness and self-reported social status remains significant. This association may reflect negatively skewed perceptions of status among lonely individuals which would be consistent with past research showing that loneliness is often accompanied by low self-esteem (Cacioppo et al., 2006), negative cognitive biases towards threats (Spithoven et al., 2017) and lower career optimism (Matthews et al., 2019). Alternatively, lower social status ratings from lonely individuals may reflect accurate perceptions of reduced ability to succeed in the labour market. Loneliness is associated with social difficulties (Knowles et al., 2015; Qualter et al., 2015) that may hinder job search activities, with qualitative evidence suggesting that research workers describe lonely individuals as nervous, awkward and likely to make poor impressions in job interviews (Matthews et al., 2022b). This may become a self-fulfilling prophecy in which lonelier individuals are less inclined to seek desirable jobs or promotions.

Our findings also shed light on the temporality of the association between loneliness and social status, with loneliness being prospectively and unidirectionally associated with social status across adolescence and young adulthood. Previous findings of loneliness both preceding (Ayalon, 2019; Morris, 2020; von Soest et al., 2020) and following unemployment and reduced subjective social status (Bu et al., 2020; Buecker et al., 2021) implied the potential for a bidirectional relationship between loneliness and social position (Morrish and Medina-Lara, 2021). However, when using longitudinal data and methods that account for stable characteristics, we did not find this to be the case for social status in young adulthood. Instead, we found that increased loneliness was longitudinally associated with reduced social status, but perceptions of social standing did not influence later loneliness. In light of the link between subjective social status and mental and physical health problems (Hoebel and Lampert, 2020), the impact of loneliness on social status may also be an additional pathway through which loneliness impacts health. Altogether, our findings underline the potential for tackling loneliness to improve socioeconomic and health outcomes as young people progress into adulthood.

Our findings should be interpreted in light of some limitations. First, as indicators of employability were not collected at age 26, it was not possible to test the direction of the association between loneliness and employability in young adulthood, constraining the conclusions that can be made about occupational and economic dimensions of social position. However, evidence suggests that the MacArthur Scale captures both material economic circumstances as well as social components of status (Galvan et al., 2023) and is associated with employment status (Shaked et al., 2016). Future research with multiple indicators of social position collected repeatedly across young adulthood could help paint a fuller picture of direction of the association between loneliness and socioeconomic position across young adulthood and help quantify the economic impact of loneliness.

Second, although the base sample was nationally representative of the British population and there was high retention up to age-18, there is evidence of differential attrition at age-26 where 73.1% of the original cohort participated, which may have provided biased results. While attrition analyses show that the age 26 sample is similar to the original sample in regard to sex (42% male, 47% at baseline), low parental SES (31%, 33% at baseline) and age 12 loneliness, some groups were disproportionately likely to not respond, in particular, participants with lower educational attainment. We used FIML methods to address missing data, which has been shown to be an effective method for longitudinal analyses with missing data and produce less biased estimates than alternatives such as listwise deletion (Enders, 2013; Widaman, 2006).

Third, while the E-Risk cohort represents the ethnic and racial composition of the UK, the relatively small number of racially minoritised participants prohibited analysis of racial and ethnic differences. Racially minoritised individuals and communities disproportionately experience economic deprivation and face increased barriers to upward social mobility which may modify the nature of the association between loneliness and socioeconomic outcomes (Ojembe et al., 2022). However, this sample is unique in being nationally representative of UK socioeconomic conditions and having collected repeated measures of loneliness across adolescence and young adulthood, such that no comparable dataset with a more diverse sample is currently available. Fourth, as this study focused on individuals living in the UK, these findings may not generalise to other national contexts. The socioeconomic conditions and class hierarchies in the UK have been shaped by particular social, political and historical factors and, as a result, do not directly map onto other national contexts. Similar research in diverse samples and in other national populations is needed to establish the degree to which the association between loneliness and socioeconomic position varies across different contexts and racialised groups.

Fifth, our results are based on data from a sample of twins and our findings may not generalise to singletons. All participants had a sibling of the same age which may shape experiences of loneliness in this sample and influence estimates of the associations between loneliness and socioeconomic outcomes. However, the extent to which being a twin could be protective against loneliness is unclear. There may, conversely, be experiences associated with being a twin that may contribute to loneliness such as being left out by peers because of assumptions that twins can rely on each other for company or being treated as part of a pair rather than as an individual. Indeed, the prevalence of loneliness in this sample is comparable to that in other samples of singletons (ONS, 2018).

Our findings have implications for researchers and policymakers. Firstly, there is a need for greater attention on the link between loneliness and socioeconomic position. Our findings indicate that reduced employability and social status are an additional burden experienced by lonely young adults and suggest that loneliness may be a force for downward social mobility. Further, in light of the link between socioeconomic status and mental health problems (Hoebel et al., 2017; Reiss, 2013), reduced socioeconomic position may be a pathway through which loneliness negatively impacts health. Longitudinal research assessing the link between loneliness, social position and health in samples further into their careers could expand understanding of the employment and socioeconomic consequences of loneliness throughout working life and assess the personal and economic costs of loneliness (Mihalopoulos et al., 2020). Our findings also point to an economic imperative for addressing loneliness for policymakers. Our results suggest that loneliness has direct costs to the economy associated with reduced employability and social position. As such, addressing loneliness may have economic benefits resulting from increased productivity and work engagement, in addition to potential indirect benefits associated with reduced healthcare burden. Individualised interventions that address loneliness in adolescence may be most effective for improving socioeconomic outcomes.

## Credit author statement

BTB: Conceptualisation, Methodology, Formal analysis, Writing – original draft, review and editing. KNT: Formal analysis Validation, Writing – review & editing. SGM: Writing – review & editing. TEM: Investigation, Writing – review & editing. CLO: Investigation, Writing – review & editing. SLSS: Writing – review & editing. MUR: Writing – review & editing. JW: Writing – review & editing. TM: Conceptualisation, Investigation, Supervision, Writing – review & editing. LA: Conceptualisation, Investigation, Supervision, Writing – review & editing.

## Funding and declaration of interests

The E-Risk Study received funding from the 10.13039/501100000265Medical Research Council (UKMRC grants G1002190 and MR/X010791). Additional support was provided by the 10.13039/100000071National Institute of Child Health and Human Development (grant HD077482) and by the 10.13039/501100003986Jacobs Foundation. Bridget T. Bryan is supported by a 10.13039/501100000856Colt Foundation PhD Fellowship. Katherine N. 10.13039/100004686Thompson is supported by an 10.13039/501100000269Economic and Social Research Council (10.13039/501100000269ESRC) LISS-DTP Studentship. The funders had no role in the study design, data collection, analysis, interpretation or writing of the report. The authors have no interests to declare.

## Transparency and data statement

The analysis plan was pre-registered at sites.duke.edu/moffittcaspiprojects/projects_2022/, and analysis code is available at github.com/bridgetbryan/loneliness-socioeconomic-consequences. The dataset analysed is not publicly available due to lack of informed consent and ethical approval but is available on request to qualified scientists. Requests require a concept paper describing the purpose of data access, ethical approval at the applicant's institution, and provision for secure data access. All data analysis scripts are available for review. For the purposes of open access, the author has applied a Creative Commons Attribution (CC BY) licence to any Accepted Author Manuscript version arising from this submission.

## Data Availability

Dataset analysed is not publicly available due to lack of informed consent and ethical approval but is available on request by qualified scientists. Link to analysis plan & code in acknowledgements.
