# Supplementary material for: The socioeconomic consequences of loneliness: Evidence from a nationally representative longitudinal study of young adults
Source: Soc Sci Med. 2024 Mar;345:116697. doi: 10.1016/j.socscimed.2024.116697 (PMC11845567; doi:10.1016/j.socscimed.2024.116697)
Supplement: Multimedia component 1 [file mmc1.docx]

**The socioeconomic consequences of loneliness:**

**evidence from a nationally representative longitudinal study of young adults**

Supplement A. Description of sample socioeconomic status

Families were recruited to represent the UK population with new-borns in the 1990s on the basis of residential location throughout England and Wales and mother’s age. Teenage mothers with twins were over-selected to replace high-risk families selectively lost to the register through non-response. Older mothers having twins via assisted reproduction were under-selected to avoid an excess of well-educated older mothers. The study sample represented the full range of socioeconomic conditions in the UK, as reflected in families’ distribution on a neighbourhood-level socioeconomic index: 25.6% of E-Risk families live in “wealthy achiever” neighbourhoods compared to 25.3% nationwide; 5.3% vs. 11.6% live in “urban prosperity” neighbourhoods; 29.6% vs. 26.9% live in “comfortably off” neighbourhoods; 13.4% vs. 13.9% live in “moderate means” neighbourhoods, and 26.1% vs. 20.7% live in “hard-pressed” neighbourhoods [ACORN: A Classification of Residential Neighbourhoods, CACI Ltd.]. E-Risk underrepresents “urban prosperity” households because they are likely to be childless. Figure S1 shows that E-Risk families’ addresses closely mirror the deciles of the UK’s 2015 Lower-layer Super Output Area (LSOA) Index of Multiple Deprivation (IMD) which averages 1,500 residents. Approximately 10% (dotted red line) of the E-Risk cohort fills each of the IMD’s 10 bands, indicating that the sample accurately represents the distribution of deprivation in the UK.


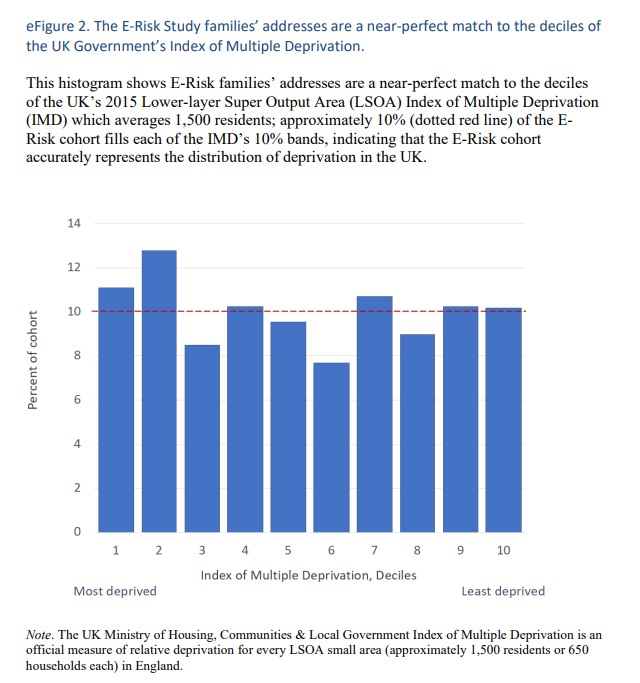
**Figure S1. Proportion of E-Risk Study family addresses in each decile of the UK Government’s Index of Multiple Deprivation.**

Note: The UK Ministry of Housing, Communities and Local Government Index of Multiple Deprivation is an official measure of relative deprivation for every LSOA small area (approximately 1,500 residents or 650 households in England).

Supplement B: Loneliness measures and means scores at each wave.

**Table S1. Cross-sectional associations of age 12 and 18 loneliness measures with important correlates**

| **Loneliness measure** | **Social isolation** | **Neuroticism** | **Anxiety** | **Depression** |
| --- | --- | --- | --- | --- |
| CDI selected items (age 12) | 0.31 | 0.17 | 0.23 | 0.22 |
| UCLA Loneliness Scale (age 18) | 0.43 | 0.27 | 0.36 | 0.41 |

*All p<0.001*.

**Table S2. Mean loneliness scores at each wave by sex and parental SES.**

| **Wave** | **Overall mean** | **Sex** | | |  | **Parental SES** | | | |
| --- | --- | --- | --- | --- | --- | --- | --- | --- | --- |
|  |  | **Male** | **Female** | ***p*** |  | **Low** | **Medium** | **High** | ***p*** |
| Age 12 | 0.64 | 0.66 | 0.61 | *0.32* |  | 0.74 | 0.65 | 0.51 | *0.001* |
| Age 18 | 1.57 | 1.51 | 1.62 | *0.22* |  | 1.69 | 1.52 | 1.49 | *0.09* |
| Age 26 | 2.43 | 2.28 | 2.55 | *0.03* |  | 2.60 | 2.25 | 2.45 | *0.40* |

Supplement C: Employability index

**Table S3. Brief description of E-Risk measures of education, employment and work-related self-perceptions, assessed at age 18 and used to compute index of employability.**

| **Measure** | **Description** |
| --- | --- |
| **Educational attainment** | Participants’ highest educational attainment was rated on a four-point scale: no qualifications (0), GCSE at grades D-G (1), GCSE at grades A*-C (2), and A Levels (3). |
| **Employment history** | Participants’ employment history was rated on a three-point scale: never employed (0), previously employed but not currently employed (1), and currently employed (2). |
| **Job preparedness: professional/technical skills** | Participants were asked whether they had any of the following skills to sell to a potential employer: word processing, computer programming, a second language, a musical or artistic talent, sales skills, customer service skills, writing skills, ‘chef’ or cooking/catering skills, repair/carpentry/plumbing/construction skills, business management, or other skills.  Positive responses were summed to create a single score indexing the number of professional/technical skills each twin believed themself to possess (mean = 4.96, SD = 1.82, range = 0 to 11, alpha = 0.64). |
| **Job preparedness:  soft skills** | Soft skills are behavioural competencies such as teamwork, decision-making, and communication that enhance an individual's interpersonal interactions, job performance and career prospects.^1^ All study members were asked whether a series of 20 phrases described them. The phrases reflected soft skills such as whether or not participants were “good at solving problems,” “a leader,” “able to explain complicated things in a simple way,” “good at communicating with others,” “able to manage time effectively,” “able to adapt well to new situations,” “someone who works well in a team,” and “able to get job advice when needed.”  Positive responses were summed to create a single score indexing the number of soft skills each twin believed him- or herself to possess (mean = 16.94, SD = 2.66, range = 0 to 20, alpha = 0.70). |
| **Career optimism** | A 10-item scale developed for the Office of Juvenile Justice and Delinquency Prevention 3-site study in the United States was used to assess participants’ optimism for their career. Participants were asked whether they agreed with statements such as “the job market is usually good to people like you,” “there is a good chance that you will make lots of money,” and “if a person like you works hard, they can get ahead.”^2^ Statements indicating pessimism about the future (e.g., “You will never have as much opportunity to succeed as other people”) were reverse scored.  Scores were summed to create a single score indexing optimism about future labour market prospects, with higher scores indicating greater optimism (mean = 16.09, SD = 3.21, range = 1 to 20, alpha = 0.68).^3^ |
| **Attitudes towards work** | Participants’ attitudes towards working were measured with 7 self-report interview questions about their behavioural and psychological commitment to working.^4^ Attitudes were assessed with the items: “If I won a lot of money on the lottery I would want to continue working,” “having a job is very important to me,” “I would get bored without a job,” “I really must have work or I will lose self-respect,” “being unemployed is one of the worst things that could happen to me,” “having a job means more to me than just the money,” and “if unemployment benefit was really high I would still prefer to work.” Possible responses included “Not true,” “Somewhat true,” and “Very true,” corresponding to scores of 0, 1, or 2.  Scores on each statement were summed to create an index of how committed the participant was to work (mean = 9.74, SD = 2.99, range = 0 to 14, alpha = 0.72); higher scores indicated more commitment.^5^ |
| **Factors hurting job chances** | Participants’ perceptions of barriers to gaining employment were assessed using 16 items. Participants were asked whether a range of demographic characteristics, personal attributes and contextual factors could hurt their chances of getting a job, including their qualifications, age, ethnicity, the economy, their personality or how they come across in an interview, how they look, their physical health or disability, a mental health or substance use problem, a criminal record, transportation problems or family commitments.  Scores were summed to create an index of barriers to employment (mean = 2.26, SD = 1.92, range = 0 to 13, alpha = 0.59). |

^1^ Secretary's Commission on Achieving Necessary Skills. What Work Requires of Schools. U.S. Department of Labor, 1991.

^2^ Huizinga D, Weiher A, Menard S, et al. Some not so boring findings from the Denver Youth Study. In: Thornberry TP, Krohn M, eds. Taking stock: An overview of findings from the Denver Youth Study. New York: Plenum Press, 1998.

^3^ Moffitt TE, Caspi A, Harrington H, Milne B. Males on the life-course-persistent and adolescence-limited antisocial pathways: Follow-up at age 26 years. *Dev Psychopathol* 2002; 14(1):179-207.

^4^ Greenberger E, O'Neil R. Spouse, parent, worker: Role commitments and role-related experiences in the construction of adults' well-being. Developmental Psychol 1993; 29:181-197.

^5^ Roberts BW, Caspi A, Moffitt TE. Work experiences and personality development in young adulthood. *J Pers Soc Psychol* 2003; 84(3):582-593.

**Figure S2. Scree plot from exploratory factor analysis on education, employment history and work-related self-perceptions items.**

**
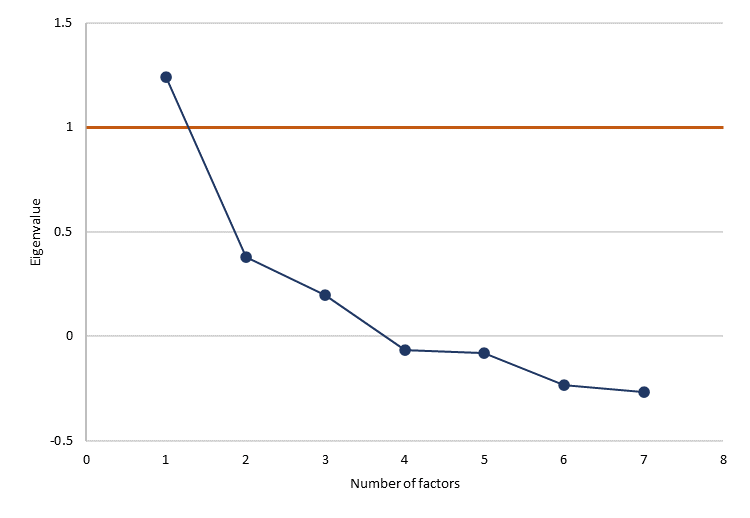
**

**Supplement D: Full RI-CLPM models and constraint testing**

To test if associations between loneliness and subjective social status were consistent over time, we constrained the autoregressive and cross-lag effects to be equal across each lag (12-18, 18-26). We tested if this equality constraint led to a significant decrease in model fit. If non-significant, associations were consistent across time points. When autoregressive and cross-lag paths were constrained, there was no loss in fit. The unconstrained and constrained models are shown in Figure S2. Chi-square fit statistics and equality constraint testing are reported in Table S2.

**Figure S3. Longitudinal association between loneliness and subjective social status across ages 12, 18 and 26 using unconstrained (A) and constrained (B) random-intercept cross-lagged panel model.
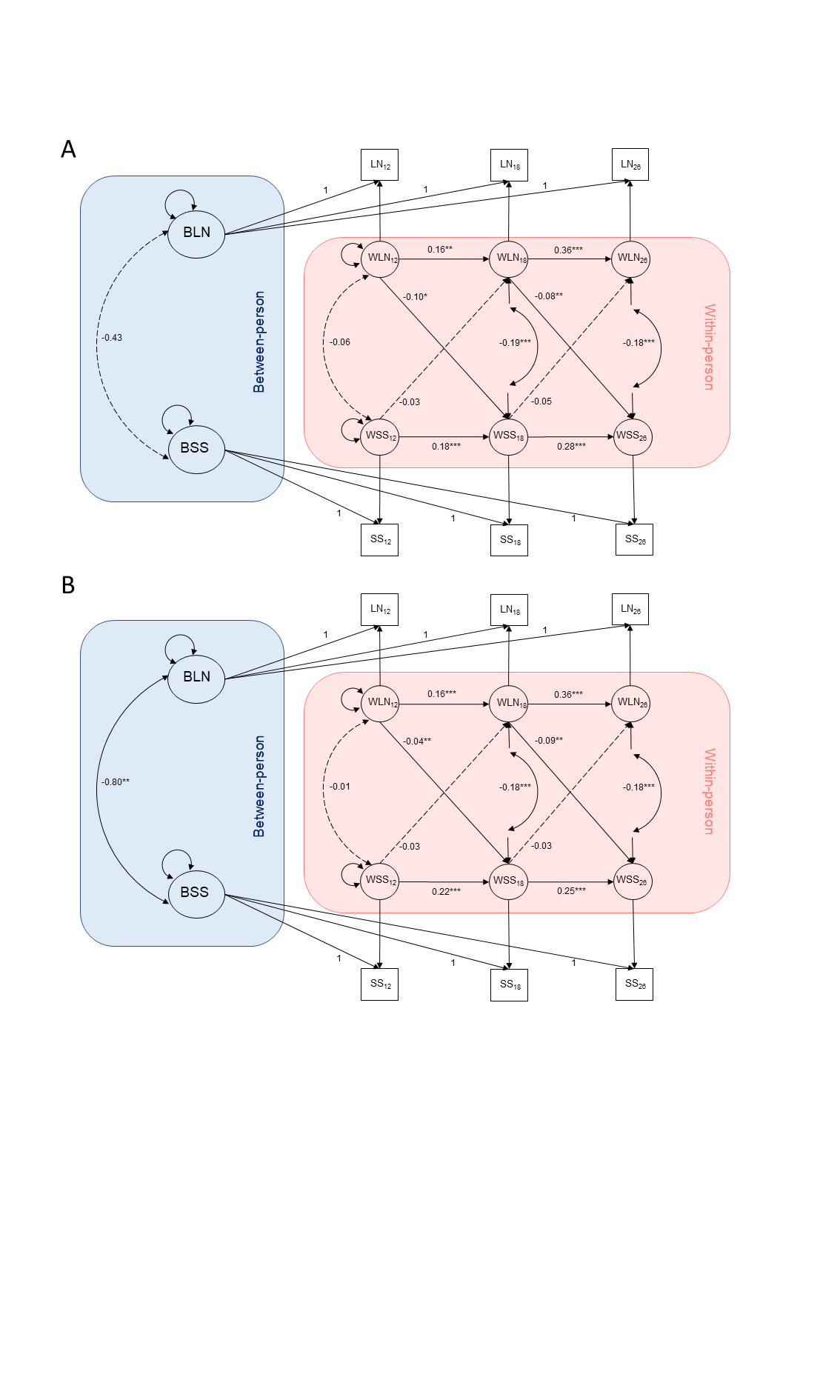
**

Note: Nonsignificant regression paths are indicated by dashed one-headed arrows. Significant regression paths are indicated by solid one-headed arrows. Correlation paths are indicated by double-headed arrows. Subscript numbers indicate time point of assessment. Within-person levels of the random-intercept cross-lagged panel models are indicated in pink, and between-person is indicated in blue. In model B, autoregressive and cross-lag paths were constrained to be equal across time. SS = measured subjective social status score; BSS = between-person level subjective social status represented by a random intercept; WSS = within-person level factor of subjective social status; LN = measured loneliness sum score; BLN = between-person loneliness represented by a random intercept; WLN = within-person level factor of loneliness.

**p < 0.05*, ** *p < 0.01, *** p < 0.001*

**Table S4. Equality constraint testing for random-intercept cross-lagged panel model (RI-CLPM) 𝝌² difference test statistics**

| **Model** | **𝝌²** | **df** | **𝝌²**$\Delta$ | **df** $\Delta$ | **p** |
| --- | --- | --- | --- | --- | --- |
| Model A: Baseline RI-CLPM | 0.79 | 1 |  |  |  |
| Model B: Constraining autoregressive and cross-lag paths | 7.69 | 5 | 6.26 | 4 | 0.18 |
